# Pharmacological Intervention in Hepatic Stellate Cell Activation and Hepatic Fibrosis

**DOI:** 10.3389/fphar.2016.00033

**Published:** 2016-02-24

**Authors:** Hans-Theo Schon, Matthias Bartneck, Erawan Borkham-Kamphorst, Jacob Nattermann, Twan Lammers, Frank Tacke, Ralf Weiskirchen

**Affiliations:** ^1^Institute of Molecular Pathobiochemistry, Experimental Gene Therapy and Clinical Chemistry, RWTH University Hospital AachenAachen, Germany; ^2^Department of Medicine III, University Hospital RWTH AachenAachen, Germany; ^3^Department of Internal Medicine I, University of BonnBonn, Germany; ^4^Department for Experimental Molecular Imaging, University Clinic and Helmholtz Institute for Biomedical Engineering, RWTH Aachen UniversityAachen, Germany

**Keywords:** liver disease, drug targeting, gene therapy, differential expression, biomedicine, antifibrotic, fibrogenesis, translational medicine

## Abstract

The activation and transdifferentiation of hepatic stellate cells (HSCs) into contractile, matrix-producing myofibroblasts (MFBs) are central events in hepatic fibrogenesis. These processes are driven by autocrine- and paracrine-acting soluble factors (i.e., cytokines and chemokines). Proof-of-concept studies of the last decades have shown that both the deactivation and removal of hepatic MFBs as well as antagonizing profibrogenic factors are in principle suitable to attenuate ongoing hepatic fibrosis. Although several drugs show potent antifibrotic activities in experimental models of hepatic fibrosis, there is presently no effective pharmaceutical intervention specifically approved for the treatment of liver fibrosis. Pharmaceutical interventions are generally hampered by insufficient supply of drugs to the diseased liver tissue and/or by adverse effects as a result of affecting non-target cells. Therefore, targeted delivery systems that bind specifically to receptors solely expressed on activated HSCs or transdifferentiated MFBs and delivery systems that can improve drug distribution to the liver in general are urgently needed. In this review, we summarize current strategies for targeted delivery of drugs to the liver and in particular to pro-fibrogenic liver cells. The applicability and efficacy of sequestering molecules, selective protein carriers, lipid-based drug vehicles, viral vectors, transcriptional targeting approaches, therapeutic liver- and HSC-specific nanoparticles, and miRNA-based strategies are discussed. Some of these delivery systems that had already been successfully tested in experimental animal models of ongoing hepatic fibrogenesis are expected to translate into clinically useful therapeutics specifically targeting HSCs.

## Characterization and Significance of Liver Fibrosis

Hepatic fibrosis is a naturally occurring wound healing reaction, driven by inflammation, which in turn is caused by persistent parenchymal injury. The main causes of hepatic damage are chronic hepatitis B and hepatitis C virus infections, alcohol abuse, biliary obstruction, autoimmune disease, hereditary haemochromatosis, and NAFLD (Ramachandran and Iredale, 2012). Untreated liver fibrosis ultimatively progresses to cirrhosis and increases the risk of developing HCC. Both hepatic fibrosis and cirrhosis are crucial causes of global morbidity and mortality (Iredale, 2007) and the incidence of advanced fibrosis in a cohort of 7000 persons was roughly estimated to be 2.8% (Poynard et al., 2010). Because there are no effective antifibrotic treatments available, there is an urgent need to develop such interventions, particularly given the progressive character and high prevalence of this disease.

The liver is made up of parenchymal and non-parenchymal cells (PCs). Hepatocytes are the only PC type, whereas KCs, LSECs, HSCs, also referred to as Ito or fat storing cells, and biliary epithelial cells belong to the group of non-PCs (Kmieć, 2001; Chen and Sun, 2011). In addition, the liver is enriched with a large variety of immune cells, including circulating intrahepatic lymphocytes and liver resident dendritic cells (Racanelli and Rehermann, 2006). In regard to hepatic fibrosis, HSCs are of particular importance, because this cell type is the source of the majority of MFBs (Povero et al., 2010). In their quiescent state, HSCs store vitamin A and retinoids, but upon tissue damage they undergo a transition to MFBs, a process that is commonly known as transdifferentiation. This process is induced by soluble mediators, including TGF-β1, PDGF, and TNF (Schon and Weiskirchen, 2014). After injury, these factors are released by hepatocytes, by activated KCs (Cubero and Nieto, 2006) and by inflammatory monocyte-derived macrophages (Zimmermann et al., 2010). The activated MFBs are highly proliferative, have the ability to migrate, and synthesize components of the ECM including collagen type I and type III, the latter being central to hepatic fibrogenesis. In this scarring process, the altered non-functional connective tissue replaces functional liver tissue. In addition to their activation by cytokines, HSCs maintain and even potentiate liver fibrosis through the effect of numerous autocrine feedback loops, thereby substantially contributing to the progression of the disease (Poelstra et al., 2012).

Similarly, portal fibroblasts that are located in portal areas are able to acquire an MFB-like phenotype and play a particularly important role in biliary fibrosis (Povero et al., 2010). Furthermore, bone marrow-derived stem cells, such as MSCs and circulating fibrocytes, can differentiate into MFBs. The cellular fraction of profibrogenic cells is further increased by hepatocytes and cholangiocytes that both have the capacity to lose their epithelial phenotype and gene expression characteristics and change into mesenchymal cells with the ability to form ECM compounds. This concept of EMT has implicated PCs as direct cellular sources contributing to matrix synthesis and fibrogenesis (Povero et al., 2010). Although EMT of cholangiocytes has been confirmed in the context of biliary fibrosis, the actual quantity of EMT of PCs in the development of liver fibrosis is still a subject of debate (Povero et al., 2010; Scholten and Weiskirchen, 2011). These somewhat contradictory observations show that activated fibroblasts in the liver are a heterogenous pool of cells (Zeisberg and Kalluri, 2013). As such, it is not surprising that the course of fibrotic disease can be so varied not only within the liver but also differ from that in other organs such as the lung, kidney, and heart, despite the obvious macroscopic and microscopic commonalities that they share. Indeed, several organ-specific features of hepatic fibrosis have been noted. While viral infections trigger hepatic fibrogenesis, infections are not a common cause for fibrosis in kidney, heart, and lung. Likewise, compared to all other organs, the liver has by far the best regenerative capacity to repair acute and chronic insults. In addition, the final outcome of fibrosis in different organs may differ dramatically. The liver is also unique with regards to the fact that advanced liver fibrosis can progress to cirrhosis and HCC (Gressner and Weiskirchen, 2006). In addition, there are several good serum markers for measurement of hepatic fibrosis, whereas biochemical blood tests that correspond for example with heart fibrosis are not available (Zeisberg and Kalluri, 2013).

Fibrosis research in hepatology employs animal models in order to detect relevant disease-promoting mechanisms and to further translate these findings into the development of antifibrotic drugs. Currently, models using rodents are the gold standard, because these models can reproduce or mimic any human liver disease independent of its cause (Liedtke et al., 2013).

At present, two models are most frequently used in experimental fibrosis (Iredale, 2007). The first model employs repetitive toxic damage such as that induced by CCl_4_. The application of this hepatotoxin creates a model of postnecrotic fibrosis (Brenner, 2009). In contrast bile duct ligation, the second model, simulates secondary biliary fibrosis (Brenner, 2009). Additional commonly used models include the use of special diets such as the methionine choline deficient diet which causes a rapid micro- and macro-vesicular steatosis resulting in liver injury similar to human NASH as well as the administration of heterologous serum, which mimics autoimmune hepatitis (Brenner, 2009). These models have been used to highlight the action of cytokine signaling pathways in liver fibrosis.

Due to their crucial role in the activation of HSCs and progression of hepatic fibrosis, cytokines, cytokine receptors, and cytokine-driven pathways offer numerous attractive therapeutic targets. Principally, there are three possibilities to target the fibrotic activities of cytokines. Firstly, a cytokine with a known antifibrotic activity can be delivered to specific target cells. Secondly, the pro-fibrogenic effect of a cytokine can be blocked by a potent inhibitor or antagonist at the site of fibrosis, thus developing an antifibrotic effect. And thirdly, cytokine receptors on specific target cells can be used as docking sites for targeted drug delivery, thereby ameliorating efficacy and reducing adverse drug reactions due to lower off-target effects. In the following, several examples that illustrate these three possibilities are given.

## Cell-Specific Delivery of Cytokines with an Antifibrotic Activity

Interleukin-10 is the prototype of an anti-inflammatory cytokine that suppresses inflammatory reactions and can attenuate excessive tissue scarring (Hammerich and Tacke, 2014). To circumvent comprehensive effects in other body regions and to evade its speedy clearance by the kidney, one research group linked IL10 to M6P-groups, which specifically bind to the M6P/insulin-like growth factor II (M6P/IGFII) receptor (Rachmawati et al., 2007). This receptor is predominantly expressed on the surface of activated HSCs during the course of liver fibrosis (de Bleser et al., 1995). The study demonstrated that the M6P-IL10 compound retained the anti-inflammatory and antifibrotic action typical of IL10, the latter indicated by a reduction of collagen type I deposition in a CCl_4_-induced rat model of experimental fibrosis. As expected, M6P-IL10 accumulated in rat livers. However, due to its negative charge, M6P does not only bind to HSCs but also to the scavenger receptor type A, which is present in KCs and sinusoidal endothelial cells (LSECs). Therefore, the modification of IL10 *via* M6P should not be considered an HSC-specific delivery, but rather a means to improve liver accumulation and pharmacokinetics, thus creating a potential candidate for therapeutic application.

Besides IL10, the cytokine IFN-γ also exhibits antifibrotic effects. IFN-γ evidently blocks different steps during the activation of HSCs as well as the synthesis of ECM in fibroblasts. Furthermore, it can even reduce fibrosis in certain patients (Rockey, 2008). Since IFN-γ has extensive pro-inflammatory properties, major problems arise in systemic therapy including adverse reactions, such as flu-like symptoms, generalized activation of immune cells, hyperlipidemia, and provocation of autoimmune reactions, and toxicity to the bone marrow and induction of depression (Bansal et al., 2011). These side effects can occur due to the fact that IFN-γ receptors are present on virtually every cell type in the body, which is likely why its antifibrotic activity is not as profound *in vivo* as it is *in vitro*. To ameliorate the benefits whilst at the same time minimizing adverse effects, it was proposed to directly deliver IFN-γ to activated HSCs. Since the platelet-derived growth factor beta receptor (PDGFβR) is abundantly found on the cell surface of activated HSCs, first a cyclic peptide specifically binding to this receptor was constructed, displaying the amino acid sequence ^∗^CSRNLIDC^∗^ as a structural element (Beljaars et al., 2003; Bansal et al., 2011). Next, this PDGFβR-recognizing cyclic peptide (PPB) was linked to IFN-γ either directly or *via* a PEG bridge. Then, the effect of both compounds on HSCs and on fibroblasts was determined *in vitro*. Finally, an *in vivo* test followed employing mice with CCl_4_-induced acute and chronic stages of liver damage. Consistently, the variant containing the PEG linker (IFN-γ-PEG-PPB) generated the most remarkable antifibrotic activity: The compound blocked both angiogenesis and hepatic inflammation and even caused fibrolysis in the advanced stage of hepatic fibrosis, while it also led to a decline of IFN-γ-associated adverse reactions (Bansal et al., 2011).

Bansal et al. (2014a,b) and coworkers further refined this approach by developing a synthetic compound consisting of the signaling portion of IFN-γ and lacking the binding site for the IFN-γ receptor, termed mim γ, and a BiPPB, both linked *via* heterobifunctional PEG adapter units. The resulting chimeric structure (mim γ-BiPPB) could solely bind to the PDGFβR on activated HSCs and considerably blocked CCl_4_-induced acute and chronic stages of hepatic fibrosis in mice as indicated by a reduction of α-SMA, desmin, and collagen type I mRNA and protein expression, while off-target effects were not observed (Bansal et al., 2014b).

## Targeted Blocking of the Pro-Fibrogenic Effect of Cytokines

Apart from PDGF, TGF-β1 is indeed the major pro-fibrogenic cytokine involved in hepatic fibrosis, as it regulates the production and deposition of ECM (Qi et al., 1999; Breitkopf et al., 2005). Generally, there are different ways for interfering with TGF-β signaling: Firstly, TGF-β expression can be down-regulated by applying anti-sense oligonucleotide mRNA, secondly, a targeted blocking of a specific isoform of TGF-β by means of monoclonal antibodies is feasible, thirdly, the activation of TGF-β receptors can be inhibited by the use of specific inhibitors, thereby halting downstream signaling, and fourthly, the local activation of TGF-β induced by α_v_β_6_ integrin and by TSP-1 can be prevented (Hayashi and Sakai, 2012).

## Tissue-Specific Blocking of the Local Activation of TGF-β

In an early study, it was established that the amino acid sequence Leu-Ser-Lys-Leu (LSKL) naturally occurs in the region of the amino terminus of the LAP and that it can hamper the activation of latent TGF-β by TSP-1 through competitive inhibition (Ribeiro et al., 1999). The LAP, which forms a dimer with mature TGF-β, is necessary for the transfer of TGF-β through the cell membrane and hampers its receptor binding before activation (Hayashi and Sakai, 2012).

To determine the influence of LSKL peptides on hepatic fibrogenesis, rats were treated with DMN for 4 weeks (Kondou et al., 2003). DMN leads to liver atrophy and fibrosis, but a concomitant daily administration of LSKL peptides significantly reduced the amount of changes compared to that observed in animals of control groups. Furthermore, the quantity of both active TGF-β1 and phosphorylated Smad2 was lower in the LSKL-treated group, indicating that LSKL blocks the activation of TGF-β1 and as a consequence the entire signaling cascade, thus avoiding the further progress of hepatic fibrosis (Kondou et al., 2003).

Later it was demonstrated that a loss of TSP-1 gives rise to a decrease of TGF-β1/Smad signaling and an increase in the proliferation of hepatocytes (Hayashi et al., 2012). Since TGF-β1 strongly blocks the proliferation of hepatocytes *in vivo* and also triggers their apoptosis, antagonizing TSP-1 down-regulates local activation of TGF-β1, up-regulates the proliferation of hepatocytes and thereby promotes liver regeneration (Hayashi et al., 2012). In contrast to its antifibrotic and regenerative effects in the liver, the lack of TSP-1 does not impact the activation of TGF-β1 during fibrogenesis in other tissues. In thrombopoietin-induced myelofibrosis as well as in bleomycin-induced pulmonary fibrosis a lack of TSP-1 does not impact bioavailability of TGF-β and does not protect from fibrosis (Evrard et al., 2011; Ezzie et al., 2011). Therefore, the local activation of TGF-β by TSP-1 seems to be tissue-specific (Hayashi and Sakai, 2012), and blocking of TSP-1, for example with LSKL peptides, and its resulting antifibrotic effect might be exclusively relevant to the liver.

## Adenovirus-Mediated Transduction of HSCs

In order to investigate the effect of overexpression of PPARγ on liver fibrosis in mice, one research group employed an adenovirus expressing PPARγ for transduction (Yu et al., 2010). PPARγ is a nuclear hormone receptor primarily occurring in the liver and in adipose tissue, which is known to regulate a multitude of genes in adipocytes (Sugii and Evans, 2011; Zhang et al., 2013). Beside endogenous fatty acids also thiazolidinediones (i.e., the glitazones), which are synthetic therapeutic agents for the treatment of type 2 diabetes, are known ligands of PPARγ (Zhang et al., 2013). With respect to hepatic fibrosis, PPARγ plays a central role in the activation of HSCs, as PPARγ expression alone is enough to restore the quiescent phenotype in activated HSCs (Hazra et al., 2004) and both activation and proliferation of HSCs correspond to exhausted PPARγ expression (Zhang et al., 2013). In mice, 2 weeks after adenoviral transduction the expression of fibrosis-associated genes markedly declined, and liver fibrosis resolved (Yu et al., 2010). Also the shift of activated HSCs to the quiescent state induced by PPARγ expression was accompanied by a sharp decline of proliferation and induction of cell cycle arrest in the G_0_/G_1_ phase as well as a rise in cell death *in vitro*, altogether leading to the conclusion that it is possible to reverse fibrosis by overexpression of PPARγ (Yu et al., 2010). In line, rosiglitazone (Avandia, GlaxoSmithKline) – a thiazolidinedione – could both reduce hepatic fibrosis in mice by downregulation of the protein expression of α-SMA, TGF-β1, and CTGF and re-establish the expression of PPARγ (Nan et al., 2009). The researchers concluded that PPARγ, induced by rosiglitazone, blocks the activation of HSCs and eliminates the expression of TGF-β1 and CTGF, thus improving liver fibrosis (Nan et al., 2009). In addition, M6P-HSA-based delivery of rosiglitazone inhibited HSC activation and diminished fibrosis in a rat model of CCl_4_-based chronic liver injury (Patel et al., 2012). However, in a large clinical trial in patients with NASH, administration of pioglitazone for 96 weeks did not significantly ameliorate hepatic fibrosis compared to placebo or a control treatment with high-dose vitamin E (Sanyal et al., 2010). Possibly, clinical data from this so called Pioglitazone *vs* Vitamin E *vs* Placebo for Treatment of Non-Diabetic Patients with Non-alcoholic Steatohepatitis (PIVENS)-trial highlight the importance of improved targeting for PPARγ agonists.

## Liposome-Mediated Delivery of IFN-α1b to Activated HSCs

A study published by Du et al. (2007) was aimed at designing an improved drug carrier that specifically targets HSCs. In preparation for the planned *in vitro* and *in vivo* experiments, they changed a cyclic peptide already characterized earlier (Marcelino and McDevitt, 1995) that had been shown to specifically recognize and bind to a non-integrin collagen type VI receptor expressed on HSCs and upregulated on activated HSCs (Beljaars et al., 2000). The amino acid sequence of the original cyclic peptide used by Beljaars et al. (2000) was Cys-Gly-Arg-Gly-Asp-Ser-Pro-Cys or C^∗^GRGDSPC^∗^, where C^∗^ indicates the cysteine residues linked *via* a disulfo bond, thus forming the ring. The modification introduced by Du and his group was the substitution of cysteine with lysine, resulting in the sequence C^∗^GRGDSPK^∗^. This cyclic peptide, which displayed a higher stability than the original one, was combined with SSLs by means of the sulfhydryl group of the cysteine residue. SSLs are liposomes chemically modified by attaching lipid derivatives of PEG, thus sterically stabilizing them (Allen et al., 1995). PEG also served as a spacer for the linkage of the cyclic peptide with the Arg-Gly-Asp motif (cRGD). The SSLs reached a diameter of around 100 nm and were long-circulating, an important basis for drug delivery *in vivo*. The experiments assessed the binding affinity of the cRGD peptide to the collagen type VI receptor on HSCs as well as determined the efficiency of delivery of IFN-α1b in rats subjected to surgical ligation of the bile duct (BDL), a common model for cholestatic fibrosis. It was shown that the cRGD peptide tended to bind to activated HSCs instead of binding to hepatocytes, that the amount of cRGD peptide-labeled liposomes (cRGD-SSLs) in HSCs extracted from BDL rats was increased by 10-fold compared to the quantity of unlabeled SSLs, and that IFN-α1b delivered in cRGD-SSLs caused a significant reduction of hepatic fibrosis in BDL rats in comparison to BDL rats of the control group or to the group of BDL rats injected with IFN-α1b delivered in unlabeled SSLs (Du et al., 2007). Blocking the proliferation of activated HSCs by boosting apoptosis, cutting ECM synthesis, and shutting down the release of TGF-β are counted among the pharmacological effects of IFN-α, responsible for the observed improvement of liver fibrosis in BDL rats (Du et al., 2007).

Taken together, this study was the first successful delivery of a drug to activated HSCs using liposomes as a carrier. cRGD-SSLs specifically bind to activated HSCs and are incorporated *via* receptor-mediated endocytosis, followed by the release of the drug. Due to their binding specificity they might qualify for targeted drug delivery to activated HSCs to improve antifibrotic therapy (Du et al., 2007). It should be noted that non-functionalized liposomes, however, do not target HSCs. In mouse models of hepatic fibrosis, dexamethasone-loaded liposomes have antifibrotic effects, but they primarily target hepatic macrophages and T-lymphocytes (Bartneck et al., 2015).

## Liposome-Mediated Delivery of Oxymatrine to HSCs

**FIGURE 1:**
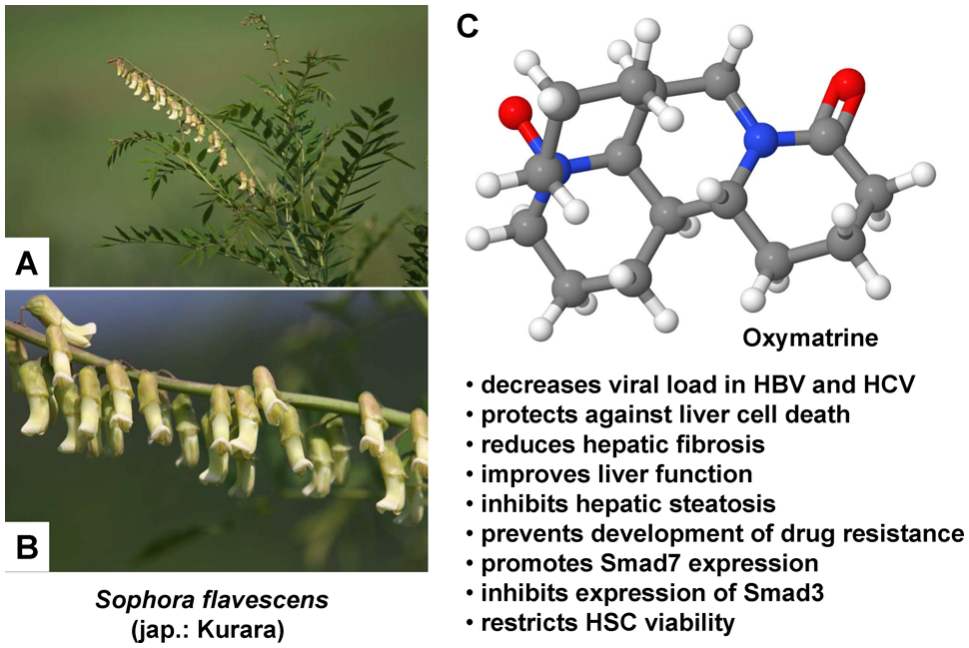
**Therapeutic potential of Oxymatrine in hepatic injury.** **(A,B)** Oxymatrine can be extracted from the roots of *Sophora* plants. *Sophora flavescens* is an evergreen, slow growing, nitrogen fixing shrub that requires a sunny site for growing. The flowers of this plant grow in simple inflorescences that vary in color from white, yellow, orange, red, or purple. **(C)** Oxymatrine is a heterocyclic quinolizidine alkaloid that has a variety of therapeutic activities in the liver. Several of these activities that were identified *in vitro* or in animal models are listed. The photos in **(A,B)** were kindly provided by Hiroshi Moriyama (http://wildplantsshimane.jp).

Chai et al. (2012) also applied RGD peptide-labeled liposomes for the specific delivery of OM in order to examine whether this herbal medicinal product would have a beneficial effect on CCl_4_-induced liver fibrosis in rats. OM is a natural quinolizidine alkaloid obtained from the roots of the *Sophora alopecuraides* L. and other *Sophora* plants (**Figure 1**), which exhibits various pharmacological properties: It blocks the replication of the hepatitis B virus (Chen et al., 2001; Wang et al., 2011) and also stops liver fibrosis (Chen et al., 2001). Besides this antiviral effect, OM additionally shows apoptosis-inducing activity, predominantly observed in several cancer cell lines, for example in human pancreatic cancer cells (Ling et al., 2011) as well as in human hepatoma SMMC-7721 (Song et al., 2006; Liu et al., 2015) and Hep-G2 cells (Liu et al., 2015). An antifibrotic effect has been studied in rats with CCl_4_-induced liver fibrosis, since OM potently limits the production as well as deposition of collagen, probably by upregulation of *SMAD7* and downregulation of *SMAD3* and *CREBBP* (CREB binding protein) gene expression, thus interfering with the canonical TGF-β1 signaling pathway (Wu et al., 2008). Before conducting their experiments, the researchers formed the OM-liposomes using lipids and the OM-containing aqueous solution and afterward coupled the RGD peptides and the OM-liposomes. Then OM-RGD-liposomes, OM-liposomes, and empty liposomes were allocated to three different groups of rats, while a fourth group of rats remained untreated. Liver fibrosis improved upon administration of OM, as assessed by reduced deposition of collagen and reduced expression of genes co-occurring with liver fibrosis, such as MMP-2, TIMP-1, and type I procollagen (Chai et al., 2012). *In vitro*, apoptosis of HSCs was induced by OM-RGD-liposomes and gene expression of *MMP2*, *TIMP1*, and *COL1A2* was inhibited. Furthermore, RGD labeling improved binding to HSCs (Chai et al., 2012).

In sum, OM improved CCl_4_-induced liver fibrosis in rats by restricting the viability of HSCs and by inducing their apoptosis. Additionally, labeling of liposomes with RGD peptides intensified binding to HSCs, thereby boosting the therapeutic benefit (Chai et al., 2012).

## Liposome-Mediated Transduction of HSCs

While most groups attempted to target HSCs and to deliver antifibrotic agents in pursuit of the goal of impairing TGF-β1-inducred collagen deposition or of eliminating activated HSCs (Adrian et al., 2007a; Sato et al., 2008; Chai et al., 2012), Narmada and coworkers, instead, focused on deploying the capacities inherent in HSCs in order to trigger liver regeneration. To that end the research group examined the antifibrotic effects of HGF production induced in HSCs (Narmada et al., 2013).

Throughout the development and differentiation of the liver in rats, HGF is mainly produced in HSCs and LSECs (Hu et al., 1993; Nakamura et al., 2011; Mogler et al., 2015). Among the various other physiological functions of HGF, suppression of apoptosis might be the most notable feature: In mice, HGF was shown to block apoptosis of hepatocytes, thus circumventing hepatic failure (Kosai et al., 1999). Contrary to this observed boost of hepatocyte survival, HGF inhibited proliferation and furthered apoptosis of α-SMA-expressing portal MFBs and activated HSCs, accompanied by resolution of cirrhosis, which was attributed to the expression of the c-Met receptor in both liver cell types (Kim et al., 2005). Furthermore, HGF countered TGF-β1-mediated production of collagen type III and α-SMA in fibroblasts (Jiang et al., 2008) and also decreased TGF-β1-induced expression of CTGF in fibroblasts in the kidney, thereby blocking synthesis of α1(I) procollagen (Inoue et al., 2003). Besides antagonizing TGF-β1-induced collagen deposition, an additional antifibrotic effect arises from its ability to trigger the expression of proteases which participate in the degradation of the ECM, including *MMP-9* (Nakamura et al., 2011), *MMP-3*, and *MMP-13* (Kanemura et al., 2008). And in addition to boosting proliferation of hepatocytes (Nakamura, 1994), HGF strengthened proliferation as well as migration of different cell types, including endothelial (Morimoto et al., 1991) and mesothelial cells (Warn et al., 2001), but in multiple cancer cell lines, such as in hepatoma HepG2 cells, HGF exerted a potent antiproliferative effect, thus obviously displaying bidirectional impacts (Tajima et al., 1991).

To target HSCs, Narmada et al. used vitamin A-coupled liposomes surrounding the HGF transgene. To that end, the researchers had to produce a plasmid containing the HGF gene, referred to as the pDsRed2-HGF plasmid. After coating of the liposomes with vitamin A, the plasmid DNA was inserted into the vitamin A-liposomes, resulting in vectors with the size of approximately 600 nm (Narmada et al., 2013). The use of vitamin A-coated liposomes as carriers addressed the ability of HSCs to take up and store vitamin A, which is why HSCs are also termed vitamin A-storing cells (Senoo et al., 2007) and the coating procedure was analogous to the one described earlier (Sato et al., 2008). It is hypothesized that vitamin A-uptake by HSCs depends on RBP and that, after forming of the vitamin A-RBP-complex, specific receptors occurring on HSCs then bind the RBP-subunit and incorporate the complex *via* receptor-mediated endocytosis (Senoo et al., 2007). But there is also evidence that RBP may not be essential for the uptake of vitamin A by HSCs: It was shown that hepatic vitamin A levels in RBP-deficient and in wild-type mice did not differ significantly, leading to the conclusion that a lack of RBP does not affect the uptake of vitamin A in the liver (Quadro et al., 1999). Moreover, vitamin A storage is not involved in profibrogenic functions of HSC *per se* (Kluwe et al., 2011). In their study Sato and colleagues could verify that vitamin A-coupled liposomes specifically target HSCs and stated that uptake *via* specific receptors for RBP would be most likely (Sato et al., 2008).

To prove the antifibrotic effects of vitamin-A-liposomes comprising the pDsRed2-HGF plasmid, Narmada et al. (2013) conducted *in vitro* investigations using a cell culture of the rat stellate cell line HSC-T6 as well as a co-culture of HSC-T6 and hepatocytes, and additionally administered the liposome formulation to rats suffering from DMN-induced hepatic fibrosis. *In vitro* experiments showed that the modified liposomes were capable of down-regulating fibrotic factors (Narmada et al., 2013). Fibrotic rats only once treated with a plasmid-containing liposomes by means of retrograde intrabiliary infusion exhibited a rise of *HGF* gene expression and a simultaneous reduction of the fibrotic markers α-SMA, TGF-β1, and collagen type I, resulting in regression of hepatic fibrosis (Narmada et al., 2013).

Taken together, targeting pDsRed2-HGF plasmid-containing vitamin A-liposomes to HSCs entailed raised *HGF* gene expression and a decline in fibrosis-specific marker proteins and could possibly be optimized to cure liver fibrosis with the help of gene therapy.

## Therapeutic Nanoparticles

Strictly taken, nanoparticles are sized from one up to 10 nm, however, the definition is frequently being extended to the sub-micron range up to 500 nm (Zhang et al., 2008). Principally, one can distinguish organic and inorganic nano-sized particulates. Gold nanoparticles are probably the most frequently used inorganic nanoparticles and are popular due to the ease of tailoring size, shape, and functionalization (Bartneck et al., 2010). Metal-based nanotherapeutics offer optical and magnetic properties, which allow their usage in imaging techniques such as computerized tomography (Bartneck et al., 2012). On the downside, inorganic carriers accumulate in the body since they are not biodegradable (Bartneck et al., 2012). Organic nanoparticles such as lipid or polymer-based constructs exhibit the advantage that many of them are biodegradable and therefore should be considered as clinically relevant nanocarriers.

Irrespective of the particle nature, its size and functionalization determine the distribution in different organs. Generally, particles or constructs sizing above 10 nm translocate to liver and spleen and only minor amounts enter the kidney, whereas those below 10 nm were shown to also enter kidney, testis, and brain (Nikoobakht et al., 2002). Nanotechnological modifications offer particle engulfment with compounds that also affect the physicochemical properties of the construct. PEG is among the most popular modifications to modify particle properties (Bartneck et al., 2010). The PEG-induced reduction of unspecific interactions with proteins also reduced uptake by liver phagocytes due to the resulting neutral particle charge (Bilzer et al., 2006).

## Targeted Delivery of Therapeutic Agents with the Help of a Cytokine Receptor

Similar to the chemical modification of cytokines, such as IL10 and IFNγ, to enable their binding to specific receptors on predefined target cells, drugs can be linked to specific carriers, allowing them to bind to cytokine receptors on target cells. With regard to liver fibrosis, the activated HSC constitutes the target cell of choice, and several efforts were carried out to selectively deliver drugs with an antifibrotic effect to activated HSCs in experimentally induced liver fibrosis.

## Targeting the M6P/IGF-II Receptor on Activated HSCs

The most frequently used receptor is the M6P/IGF-II receptor, which is a transmembrane glycoprotein with a wide range of regulatory functions. Due to its structure, it can act as a clearance receptor, leading to degradation of proteins through endocytosis and as a signaling receptor engaged in G-protein dependent signal transduction (El-Shewy and Luttrell, 2009; Heger and Schlüter, 2013). Based on the large extracellular portion of the M6P/IGF-II receptor, binding of many different ligands is possible: Both interactions with M6P-containing ligands, such as renin, latent TGF-β1, thyroglobulin, proliferin, leukemia inhibitory factor, and granzyme B, and recognition of M6P-free ligands, for example IGF-II, retinoic acid, urokinase-type plasminogen activator receptor, and plasminogen are well documented (Heger and Schlüter, 2013). The cytoplasmic domain does not display any catalytic activity, so after ligand binding the receptor shuttles between the cell membrane and intracellular compartments, such as lysosomes (El-Shewy and Luttrell, 2009; Heger and Schlüter, 2013).

**FIGURE 2:**
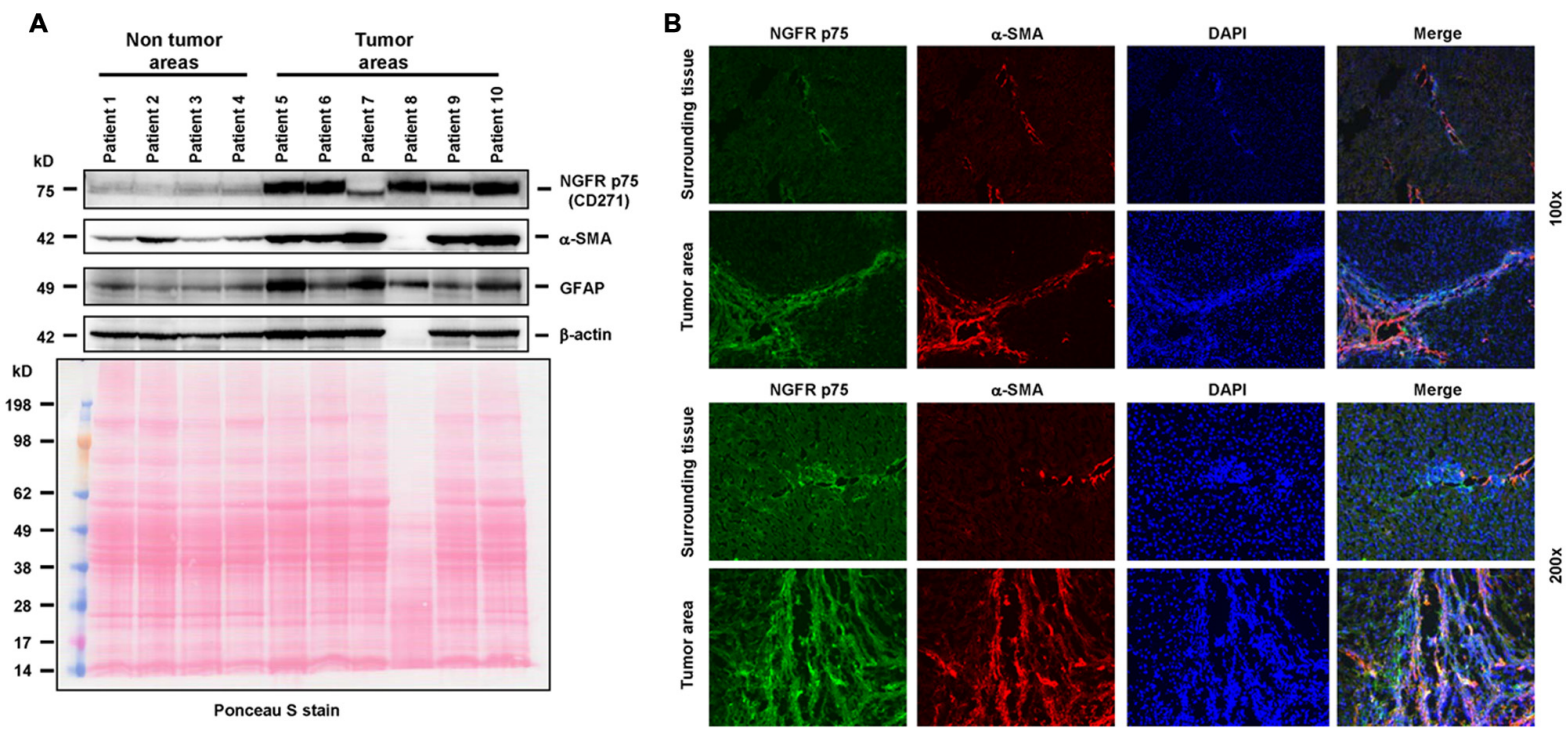
**The neutrotrophin receptor as a therapeutic target.** **(A)** The tyrosine kinase NGFR p75 is significantly upregulated in HSCs during cancerogenesis. In this experiment, the expression of NGFR p75 was analyzed by Western blot in human specimens derived from non-tumor and tumor areas of HCC patients. The analysis revealed a strong upregulation of NGFRp75 in diseased liver areas. The following antibodies were used in this set of experiments: NGFR p75 (sc-6188, Santa Cruz Biotech, Santa Cruz, CA, USA), α-SMA (CBL171, Cymbus Biotech, Hampshire, UK), glial fibrilliary acidic protein (GFAP, sc-6170, Santa Cruz), β-actin (A5441, Sigma, Taufkirchen, Germany). The membrane was counterstained with Ponceau S to document equal gel loading. The striking difference in protein content in sample “patient 8” was due to extremely high bilirubin content in this probe. **(B)** Immunhistochemical analysis of NGFR p75 and α-SMA expression in human samples obtained from normal and tumorigenic liver samples. Please note the strong expression of NGFR p75 in cells that were stained positive for α-SMA that becomes visible in the merged images. Cells were counterstained with DAPI. Magnifications were 100x (*upper two panels*) or 200x (*lower two panels*). The usage of human samples is covered by an ethical vote from the relevant authority (Institutional Review Board of the Bonn University Ethics Committee, decision #067/10). The results depicted in this figure are similar to a variety of previous studies that have already shown that NGFR p75 is expressed by HSC and strongly upregulated during transdifferentiation (Trim et al., 2000; Cassiman et al., 2001; Asai et al., 2006; Passino et al., 2007; Kendall et al., 2009; Zvibel et al., 2010; Reetz et al., 2013; Patil et al., 2014).

The first step on the path to targeted delivery was made by Beljaars et al. (1999), when they developed and tested a carrier, designed to specifically bind to the M6P/IGF-II receptor on activated HSCs. This receptor is strongly activated during transdifferention of HSC and is closely associated with expression of α-SMA and the pathophysiology of liver disease (Trim et al., 2000; Cassiman et al., 2001). For example, it is strongly upregulated in HSC during hepatic carcinogenesis (**Figure 2**).

**FIGURE 3:**
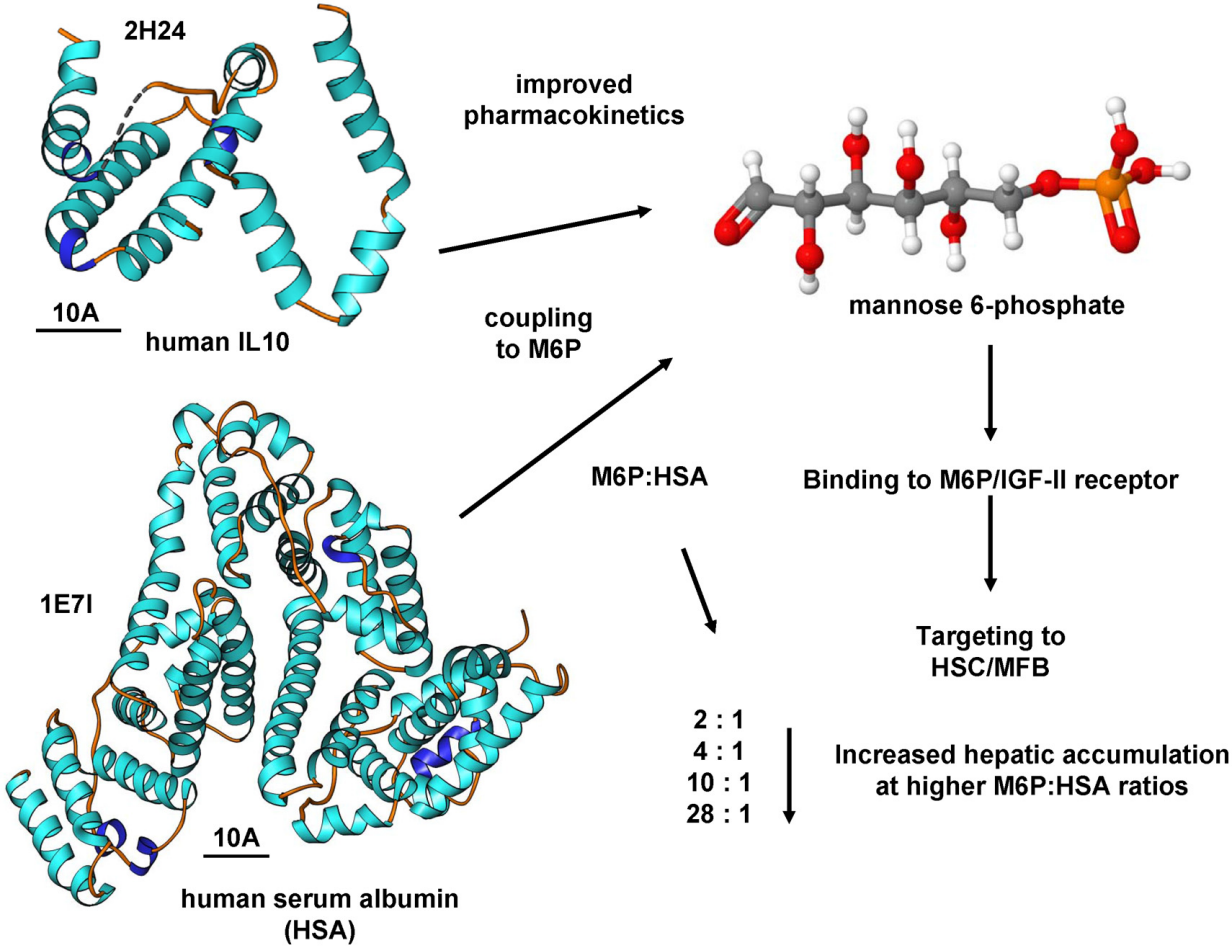
**Targeting the M6P/IGF-II receptor by M6P coupling.** The coupling of substances to mannose-6-phosphate (M6P) is an effective means to increase the activity of the M6P/IGF-II receptor. The coupling of human serum albumin resulted in improved pharmacokinetics and affinity for the M6P/IGF-II receptor. This receptor is significantly increased in profibrogenic cells expressing α-SMA. High MP6:HSA coupling ratios resulted in increased hepatic accumulation (Beljaars et al., 1999). This concept of M6P coupling was also successfully tested for IL10 (Rachmawati et al., 2007). The structures of human IL10 and HSCs were generated using the entries 2H24 and 1E7I that are deposited in the RCSB Protein Data Bank and the Ribbons XP software. The space bars each represent 10A. Details about the crystal structures of human IL10 and HSA are provided elsewhere (Bhattacharya et al., 2000; Yoon et al., 2006).

The mentioned carrier consisted of HSA, to which M6P groups had been added: Four different constructs with an increasing amount of M6P groups, i.e., with a molar ratio of M6P:HSA amounting to 2:1, 4:1, 10:1, and 28:1, were generated (Beljaars et al., 1999). First, the accumulation of the compounds in fibrotic livers of rats was determined: the uptake rose with an increasing degree of substitution (**Figure 3**). Compounds with a molar ratio less than or equal to 10:1 accumulated only at rates of 9% ± 5% or less in the liver, whereas a ratio of 28:1 led to a jump to 59% ± 9% of the originally delivered dose (Beljaars et al., 1999). Next, the accumulation in HSCs was ascertained employing double-immunostaining techniques, i.e., for HSA and HSCs: Here, too, the uptake of M6P-HSA rose with an increasing degree of substitution and 70% ± 11% of the intrahepatic staining for the conjugate with a 28:1 ratio was located in HSCs (Beljaars et al., 1999). In addition, it could also be shown that BSA linked to M6P massed in human non-PCs of the liver (Beljaars et al., 1999). The authors concluded that M6P-albumins could function as selective drug carriers to HSCs.

Since then, the M6P-HSA carrier has been used more often for the delivery of antifibrotic drugs to HSCs. The immunosuppressive drug MPA, which is capable of blocking proliferation of fibroblasts, was coupled to M6P-HSA, intending to avoid immunosuppression and to improve its antifibrotic activity (Greupink et al., 2005). Specific binding of the conjugate to HSCs and a reduction of their proliferation could be observed *in vitro*. *In vivo*, it selectively accumulates in the liver – primarily in HSCs and also in KCs and LSECs, but not in primary and secondary lymphoid tissues (Greupink et al., 2005). Furthermore, in BDL treated rats the compound minimized liver inflammation and, likewise, the mRNA expression of α-β-Crystallin, which functions as a marker for the activation of HSCs (Greupink et al., 2005). In summary, coupling of MPA to the M6P-HSA carrier and subsequent targeted delivery led to a decline of the activation of HSCs.

Using the same carrier, the antifibrotic drugs PTX, a drug that has an antiproliferative effect on HSCs and blocks their activation *in vitro* (Gonzalo et al., 2006), DOX, which also potently impairs proliferation of HSCs *in vitro* (Greupink et al., 2006), 15-deoxy-Δ12,14-prostaglandin J2 (15dPGJ2), an apoptosis-inducing drug (Hagens et al., 2007), as well as 4-chloro-N-[4-methyl-3-(4-pyridin-3-yl-pyrimidin-2-ylamino)-phenyl]-benzamide (PAP19), a tyrosine kinase inhibitor and a derivative of imatinib (Gonzalo et al., 2007) and 18β-glycyrrhetinic acid (18β-GA) also known as enoxolone (Luk et al., 2007) could all be successfully delivered to HSCs to selectively unfold their respective activity.

Another research group applied a M6P-BSA carrier in a slightly different form: The researchers coupled TFOs and M6P-BSA with the help of disulfide bonds (Ye et al., 2005). TFOs can be used for gene silencing through triplex formation with genomic DNA, i.e., promoter sequences, thereby inhibiting transcription of the genes concerned. To optimize the uptake of M6P-BSA-TFO by the liver, based on prior testing a conjugate with a molar ratio of M6P:BSA equal to 20:1 was chosen. It could be determined that nearly 66% of the administered conjugate had accumulated in the liver of rats 30 min following its injection, and a high amount of the delivered dose could be detected in HSCs, thus suggesting its use for the therapy of liver fibrosis (Ye et al., 2005).

Adrian et al. (2007b) described the coupling of M6P-HSA to liposomes with the aim to ascertain the pharmacokinetics of M6P-HSA-liposomes and their specificity to target HSCs. Liposomes constitute artificial globular entities with one or more phospholipid bilayers forming an envelope around an aqueous core (Giannitrapani et al., 2014; Neophytou and Constantinou, 2015) and are capable of carrying both hydrophilic and lipophilic agents: Hydrophilic drugs are placed within the aqueous core whereas lipophilic molecules are integrated into the phospholipid bilayer. Being counted among the organic nanoparticles, liposomes feature several advantages: Beyond their solid biocompatibility they can be easily produced, exhibit only minor systemic toxicity, and are quickly eliminated from the blood and taken up by the reticuloendothelial system (Giannitrapani et al., 2014). In preparation for their study, the researchers added 29 M6P groups on average to each albumin molecule and, in a second step, about 31 M6P-HSA molecules on average were linked to one liposome. The resulting M6P-HSA-liposomes had a size of roughly 100 nm. BDL-treated rats were employed to perform the following *in vivo* experiments: Radioactively labeled M6P-HSA-liposomes was injected intravenously, and both their elimination from the blood as well as their distribution were monitored. Ten minutes following injection, 90% of the original dose of ^3^[H]-M6P-HSA-liposomes was removed from the blood and predominantly aggregated in the liver (Adrian et al., 2007b). Apart from HSCs M6P-HSA-liposomes also concentrated in KCs and LSECs, and accumulation in the last two cell types could be blocked by previously administered polyinosinic acid, which acts as a competitive inhibitor of scavenger receptors class A, indicating that this receptor type is responsible for the recognition of M6P-HSA by KCs and LSECs and not by HSCs (Adrian et al., 2007b). Furthermore, injection of uncombined M6P-HSA prior to the administration of M6P-HSA-liposomes resulted in a decreased liver uptake of 15% accompanied by an increase of accumulation in the lungs, due to an extended presence in the blood circulation and probably binding to pulmonary intravascular macrophages within the injured lungs of BDL rats.

**FIGURE 4:**
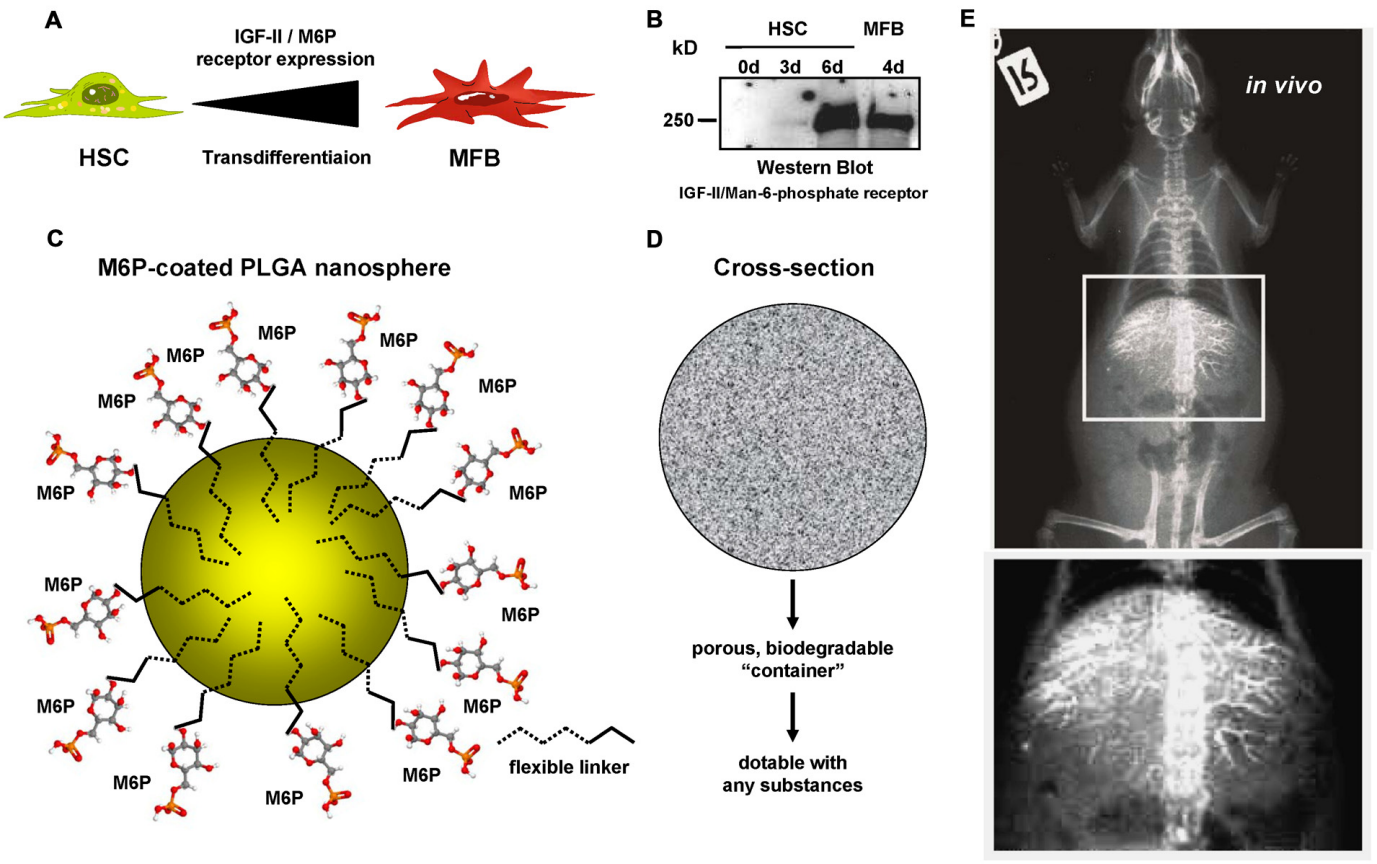
**Mannose-6-Phosphate coating of nanoparticles for targeted delivery of pharmacological active compounds to activated HSCs and MFBs.** **(A)** Several reports have shown that the expression of the IGF-II/M6P receptor is strongly upregulated during transdifferentiation of quiescent HSCs to MFBs. **(B)** Western blot analysis of cellular protein extracts of rat HSCs and rat MFBs that were cultured for indicated time intervals. The blot was probed with a goat-anti-IGFIIR (sc-14413, Santa Cruz Biotechnology, Santa Cruz, CA, USA). **(C)** In a former study, we have generated biocompatible nanoparticles that are based on PLGA and coated with M6P *via* flexible linkers. **(D)** These biodegradable particles have a porous container structure allowing to pack up (“dotting”) various compounds into the cavities. **(E)** Respective nanoparticles were dotted with barium sulfate and injected into the tail vein of a rat. The radiography, that was prepared approximately 2 h later, shows that these particles were enriched in the liver. More details about the production of the modified PLGA particles used in this study can be found elsewhere (Pereira Paz, 2004).

**FIGURE 5:**
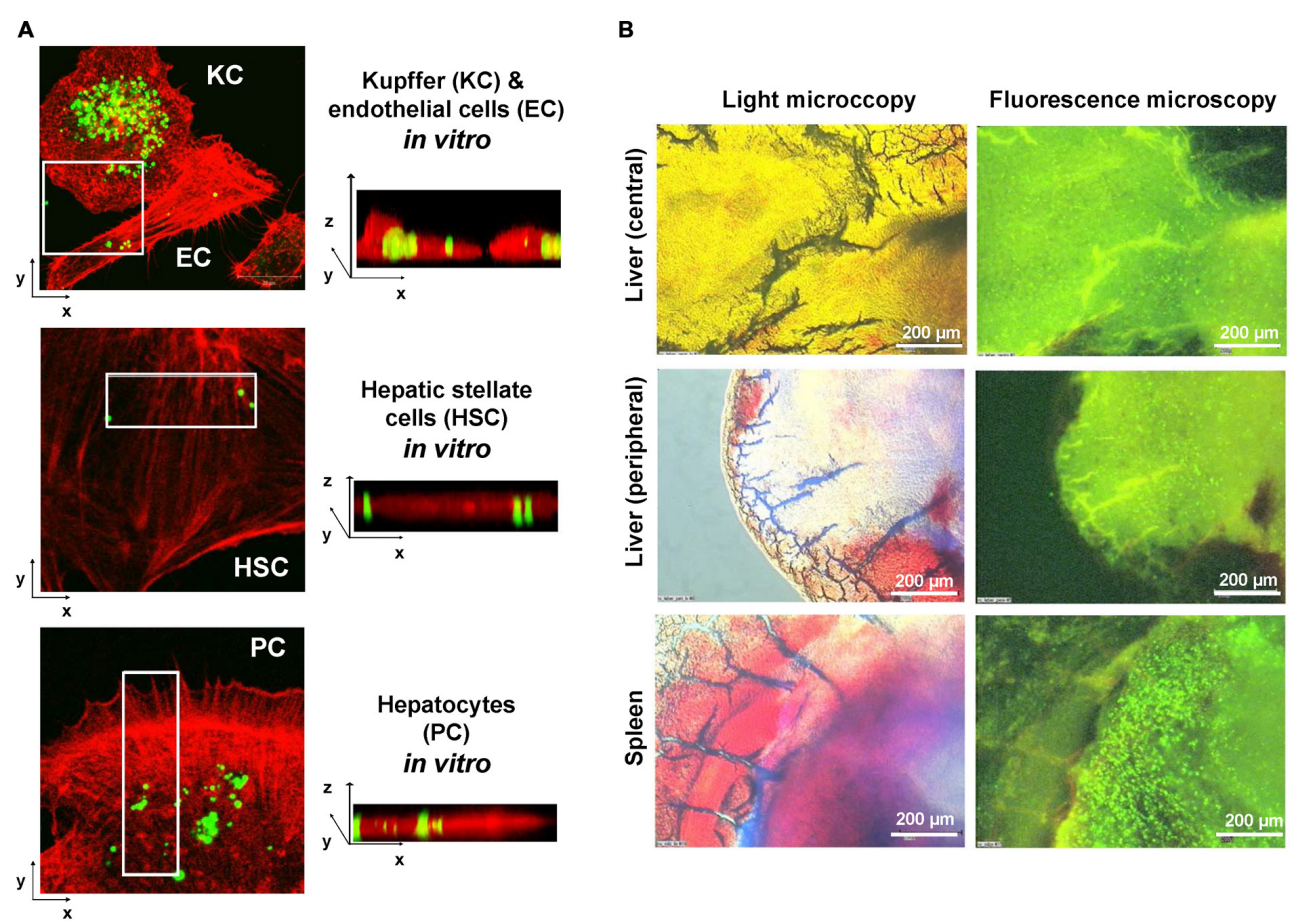
**Limitation of the M6P targeting strategy.** **(A)** Primary isolated KC, endothelial cells (EC), HSCs, and hepatocytes (parenchymal cells, PC) were incubated with FITC-dextran-doted M6P-coated nanospheres. After 24 h, excess nanospheres were removed by washing two times with phosphate-buffered saline. The growth medium was renewed and cells further incubated for another 24 h period. Then the cells were fixed in 4% paraformaldehyde and the cellular actin was stained with a phalloidin-rhodamine complex. After a further wash step, the cells were fixed in Moviol (Merck Biosciences, Bad Soden, Germany) and analyzed by confocal microscopy. **(B)** FITC-dextran doted nanospheres were injected *via* the tail vein into a stunned rat. 24 h later, the animal was sacrificed and organ specimen derived from the central or peripheral area of the liver as well as from the spleen analyzed by light microscopy (left panels) and fluorescence microscopy (right panels). Please note the high concentration of nanospheres (green beads) within the spleen. More details about this set of experiments can be found elsewhere (Benner, 2007).

In previous studies (Pereira Paz, 2004; Benner, 2007), we in cooperation with other groups have established novel biodegradable M6P-coupled PLGA nanoparticles that have a porous structure allowing to be dotted with various substances (**Figure 4**). Based on the finding that the IGF-II/M6P receptor is found to be strongly upregulated during transdifferentiation, we tested if these vehicles can be targeted into the liver. Therefore, we dotted these nanoparticles with barium sulfate that acts as a radiopaque contrast media and found that respective nanoparticles were enriched in the liver when applied *via* the tail vein (cf. **Figure 4**). Unfortunately, in our hands the synthesized particles caused a serious pulmonary embolism that prevented further exploration of these biodegradable vehicles. In addition, when we tested their specificity *in vitro*, we found that respective FITC-dextran-loaded particles were taken up not only by HSCs but also by ECs and hepatocytes and highly effective by KCs (**Figure 5A**). This finding was more or less independent from their surface loading with M6P (not shown), possibly indicating the exceptional scavenging ability of the cells. Likewise, the application of FITC-dextran-loaded nanospheres *in vivo* resulted in a strong uptake of the respective particles not only in the liver but also in the spleen (**Figure 5B**).

In sum, M6P-HSA-liposomes might be suitable to specifically deliver drugs to HSCs, LSECs and KCs, and both M6P/IGF-II and scavenger receptors have the ability to detect and bind the M6P-HSA subunit (Adrian et al., 2007b).

## Targeting the p75 Neurotrophin Receptor on HSCs

Aiming at developing an effective strategy to specifically target HSCs using a gene delivery vector, Reetz et al. (2013) modified the Ad serotype 5 envelope by two frequently used strategies for *in vivo* targeting and evaluated the transduction efficiency of both procedures. Since the wild-type Ad is capable of infecting a wide spectrum of different cell types in the liver, such as hepatocytes, KCs and HSCs, its application for cell-specific infection is challenging. This ubiquitous tropism is caused by the modality of viral entry into the cell. First, Ad fiber knobs interact with receptors on the surface of a host cell, particularly with the CAR, thereby attaching the virus to the cell surface, followed by a further interaction of the virus-CAR-complex with other co-receptors, as for instance various integrins (Sharma et al., 2009; Reetz et al., 2013). An RGD amino acid sequence within the penton base, i.e., the coat protein of the Ad, is responsible for binding to the vitronectin receptors αvβ3 and αvβ5, whereupon *via* integrin-mediated signaling the internalization of the Ad is effected by means of receptor-ediated endocytosis (Wickham et al., 1995; Sharma et al., 2009). Since the virus-CAR interaction features high affinity in contrast to the rather low affinity of the virus-integrin interaction, the former is critical for the efficiency of infection and the tissue-specific occurrence of the CAR is one of the key factors which determine adenoviral tropism (Sharma et al., 2009). In consequence, to restrict the natural tropism of the Ad it is necessary to sidestep binding of the virus to the primary receptor and, instead, redirect the virion to a receptor solely expressed on the target cell, which should be genetically modified. To put this approach into practice Reetz et al. (2013) developed a peptide (NGF_P_) derived from the NGF and specifically binding to the p75 NT receptor (p75NTR) expressed on HSCs. Being part of the TNF superfamily, p75NTR is structurally characterized by a cysteine repeat motif, which acts as a ligand binding domain and a cytoplasmic portion called the death domain (Casaccia-Bonnefil et al., 1999). Ligands of p75NTR are the NTs with NGF as prototype (Roux and Barker, 2002), as well as BDNF, NT-3, and NT-4, which are relevant for the development of the nervous system in vertebrates (Lee et al., 2001). Additionally, NTs can also initiate signaling by binding to Trk receptors with tyrosine kinase activity and, depending on the participating receptors, signal response can vary. If both p75NTR and Trk-A are stimulated, cell survival will be triggered, whereas exclusive interaction with p75NTR induces cellular death (Trim et al., 2000; Roux and Barker, 2002). The fact that p75NTR is expressed by HSCs and that NGF-binding to p75NTR induces apoptosis of activated HSCs suggested that stimulation of p75NTR could selectively target and eliminate HSCs (Trim et al., 2000).

**FIGURE 6:**
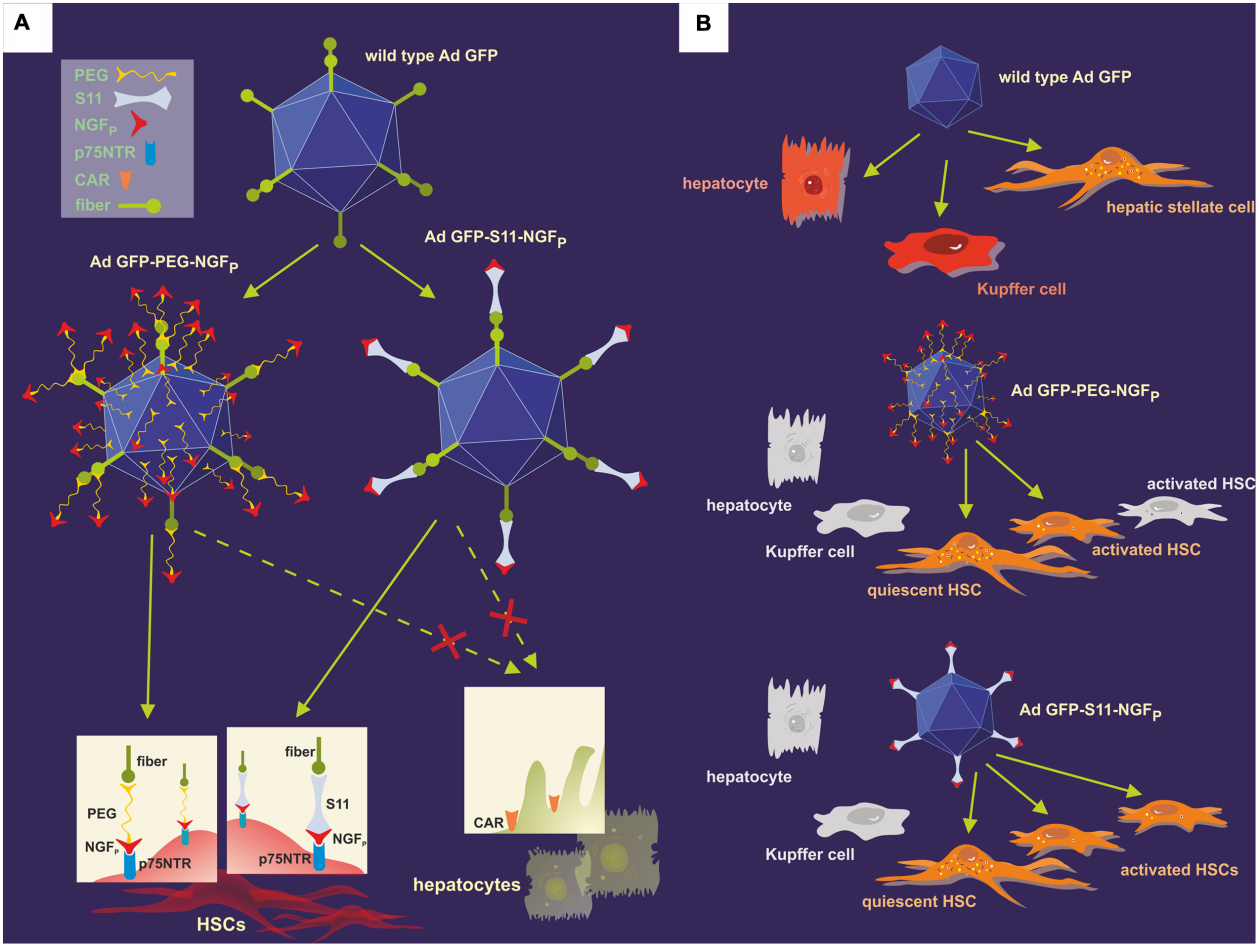
**Modification of the natural adenoviral tropism employing two different approaches resulted in the specific targeting of the p75NTR on HSCs.** **(A)** The p75NTR-binding peptide NGF_P_ and the wild-type Ad.GFP were covalently joined using PEGylation, thus forming Ad.GFP-PEG-NGF_P_ (left) as well as merged with the help of S11 adapter molecules, resulting in Ad.GFP-S11-NGF_P_ (right). The single chemical bonds of PEG and viral proteins are close together, thereby concealing both fiber knobs and RGD sequences on the viral surface, whereas the S11 fragment only binds to the fiber knobs, keeping the RGD sequences free for integrin interaction. Since fiber knobs are blocked in either case, binding to CAR, for example on hepatocytes, is inhibited and exclusive interaction of NGF_P_ with p75NTR on the surface of HSCs can occur. **(B)** Wild-type Ad.GFP vectors can transduce hepatocytes, Kupffer cells, and HSCs in the liver, identifiable by the green fluorescence of GFP (top). As a result of the viral modifications, both Ad.GFP-PEG-NGF_P_ (middle) and Ad.GFP-S11-NGF_P_ (below) exclusively bound to and transfected quiescent and activated HSCs, but Ad.GFP-S11-NGF_P_ showed an enhanced transduction efficiency concerning activated HSCs (below).

More than a decade later, Reetz et al. (2013) employed this concept to demonstrate the adenovirus-mediated transfection of HSCs for the first time. In their experiments, the researchers employed a wild-type Ad serotype 5 (Ad.GFP) as the vector, transferring the GFP gene and retaining the adenoviral natural tropism (**Figure 6**). The synthetic NGF_P_, composed of amino acids 25–36 of the original NGF protein and exactly corresponding to the portion of NGF necessary for binding to p75NTR, was then coupled to Ad.GFP using two different techniques in order to vary the viral tropism: A single chain antibody fragment (S11), specifically recognizing the Ad fiber knobs as well as NGF_P_, served as an adapter molecule to form Ad.GFP-S11-NGF_P_. Furthermore, NGF_P_ and Ad.GFP were covalently joined by means of polyethylene glycol – a procedure termed PEGylation – thus building Ad.GFP-PEG-NGF_P_. Subsequently, both derivatives were tested *in vitro* and *in vivo*, allowing determining the efficiency of transfection based on the level of green fluorescence. Additionally, in healthy and in BDL-treated mice HSCs were detected on the basis of their ultraviolet vitamin A-fluorescence, as recorded by intravital fluorescence microscopy. Data demonstrated that usage of both Ad.GFP-S11-NGF_P_ and Ad.GFP-PEG-NGF_P_ restricted liver tropism characteristic of the wild-type Ad, enlarged transfection of HSCs, and both vectors equally displayed higher transduction efficiency in fibrotic liver versus healthy organs, but – compared with Ad.GFP-PEG-NGF_P_ – Ad.GFP-S11-NGF_P_ showed a higher targeting efficiency of activated HSCs (Reetz et al., 2013).

In conclusion, targeting of p75NTR on HSCs with the use of a modified adenoviral carrier could constitute a practicable and powerful way of applying gene therapy to activated HSCs (Reetz et al., 2013).

## Targeting PDGF and the RBP Receptors on HSCs

PDGF is the most mitogenic factor that leads to increased HSC proliferation in liver fibrosis. PDGFRβ, which is one of its two receptors, is critically upregulated on activated HSCs (Bonner, 2004). Encouraging studies revealed a sterically-stabilized liposome (SSL) equipped with the cyclic peptide “C^∗^SRNLIDC^∗^ (pPB) with strong binding activity for the PDGF-β receptor (and loaded with IFN-γ) enhanced anti-fibrotic effects of IFN-γ in a murine model of TAA-based hepatic fibrosis (Li et al., 2012).

**FIGURE 7:**
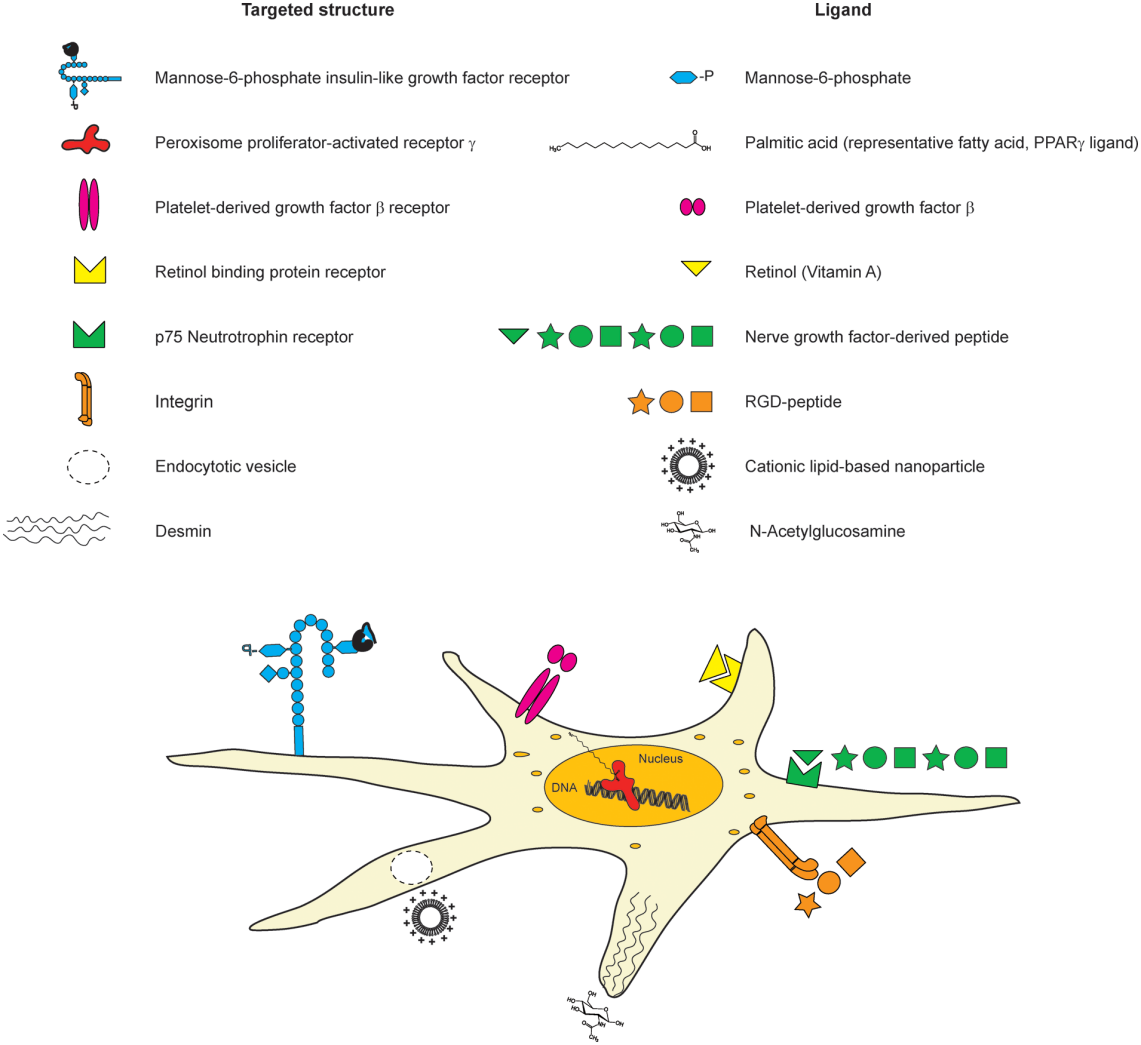
**Cellular structures for targeting HSCs with nanoconjugate ligands.** There are diverse different structures on HSCs, which have been used earlier for targeted delivery. The targeted structures (list on the left side), the corresponding ligands (right side list), and their location on HSCs are depicted in the scheme.

The retinol binding protein receptor which is involved in storing retinol is another putative structure for targeting HSCs. Hence, lipid-encapsulated and retinol-decorated siRNA versus heat-shock protein 47, a collagen-specific chaperone, was shown to ameliorate fibrosis in diverse experimental mouse models (Sato et al., 2008). Nanoconjugate siRNA against TGF-β1 equipped with an *N*-acetylglucosamin targeting moiety intending to reach HSCs *via* desmin was reported to colocalize with HSCs and to reduce fibrosis (Kim et al., 2013). Due to the cytosolic presence of desmin, the conjugates may also enter the cells *via* alternative routes. Recently, siRNA directed against the procollagen α1(I) gene has been deployed using cationic nanoparticles, leading to an amelioration of fibrosis. However, despite the therapeutic success in preclinical studies, the delivery route was rather unspecific and probably occurs through endocytosis since none of the known cell-targeting motifs were used (Jiménez Calvente et al., 2015) (**Figure 7**).

## Transcriptional Targeting Approaches for Activated HSCs/MFBs

Comparative mRNA and protein expression analyses demonstrated that HSCs have a characteristic and unique gene signature that is significantly modulated during the transdifferentiation into MFBs (Kristensen et al., 2000; Ji et al., 2012; Azimifar et al., 2014; Zhang et al., 2015). Therefore, it was assumed that several of these genes might be attractive targets for tracking, targeting and cell isolation (Zhang et al., 2015). There is a growing list of genes that are either expressed preferentially in HSCs (within the liver) or become significantly upregulated in response to cellular activation and transdifferentiation. The group of “transdifferentiation-sensitive genes” include transcription factors, ECM proteins, cell adhesion molecules, smooth muscle specific genes, cytokine receptors, and genes encoding proteins involved in matrix remodeling, or cytoskeletal organization (**Table 1**).

**Table 1:** Selected genes preferentially expressed in HSCs or showing transdifferentiation dependent activation in activated HSCs/MFBs.

Gene	Experimental finding	Reference
*ABCC9* (SUR2, ATP-binding cassette, subfamily C, member 9)	Identified by proteomic functional comparative hotspot analysis of liver cells for genes associated with ECM assembly	Azimifar et al., 2014
*ACTA2* (α-smooth muscle actin)	α-smooth muscle actin increases during prolonged culturing in rat HSCs	Ramadori et al., 1990; Kristensen et al., 2000^∗^
*ACTG1* (γ actin)	Activated during *in vitro* and *in vivo* transdifferentiation	Kristensen et al., 2000
*ADAMTSL2* (ADAMTS-like protein 2)	Identified by proteomic functional comparative hotspot analysis of liver cells for genes associated with ECM assembly	Azimifar et al., 2014
*ANGPTL2* (Angiopoietin-like Protein 2)	Identified by proteomic functional comparative hotspot analysis of liver cells for genes associated with ECM assembly	Azimifar et al., 2014
*ANGPTL6* (AGF, Angiopoietin-like Protein 6)	Identified by proteomic functional comparative hotspot analysis of liver cells for genes associated with ECM assembly	Azimifar et al., 2014
*ARP3-β* (Actin-related protein 3-β)	Identified by proteomic functional comparative hotspot analysis of liver cells for genes associated with ECM assembly	Azimifar et al., 2014
*BAG2* (Bcl2-associated athanogene 2)	Up-regulated in rat HSCs during activation process	Ji et al., 2012^∗∗^
*BAG3* (Bcl2-associated athanogene 3)	Up-regulated in rat HSCs during activation process	Ji et al., 2012
*BDNF* (brain-derived neurotrophic factor)	HSCs show immunoreactivity for the brain-derived neurotrophin	Cassiman et al., 2001
*BGN* (biglycan)	The steady-state levels of the mRNA for biglycan increased threefold during primary HSC culture	Meyer et al., 1992;Ji et al., 2012
*C1S* (Complement component 1s, Serine protease)	Activated during *in vitro* and *in vivo* transdifferentiation	Kristensen et al., 2000
*CALB3* (Calbindin D9k)	Identified by proteomic functional comparative hotspot analysis of liver cells for genes associated with ECM assembly	Azimifar et al., 2014
*CALD1* (h-caldesmon)	Activated rat HSCs express this smooth muscle cell marker	Wirz et al., 2008
*CAPN6* (Calpain 6)	Identified by proteomic functional comparative hotspot analysis of liver cells for genes associated with ECM assembly	Azimifar et al., 2014
*CAPZA1* (F-actin capping protein α1)	Activated during *in vitro* and *in vivo* transdifferentiation	Kristensen et al., 2000
*CD47* (IAP, integrin-associated protein)	Identified by proteomic functional comparative hotspot analysis of liver cells for genes associated with ECM assembly	Azimifar et al., 2014
*CD73* (NT5E, ecto-5′-nucleotidase)	CD73 is weakly expressed in quiescent HSCs and portal fibroblasts but is markedly upregulated at the transcriptional level in myofibroblastic HSCs and portal fibroblasts	Fausther et al., 2012
*CD276* (B7H3. B7 homolog 3)	Up-regulated in rat HSCs during activation process	Ji et al., 2012
*CNN1* (Calponin H1)	Activated rat HSCs express this smooth muscle cell marker	Wirz et al., 2008
*COCH* (Cochlin)	Identified by proteomic functional comparative hotspot analysis of liver cells for genes associated with ECM assembly	Azimifar et al., 2014
*COL1A1* (Collagen α1 (I))	The rate of collagen synthesis by HSCs isolated from CCl_4_-treated rats is four- to sixfold higher than in HSCs isolated from untreated control animals. The gene is activated during *in vitro* and *in vivo* transdifferentiation	Shiratori et al., 1987; Kristensen et al., 2000
*COL1A2* (Collagen α2 (I) c-terminal propeptide)	Activated during *in vitro* and *in vivo* transdifferentiation	Kristensen et al., 2000
*COL3A1* (Collagen α1 (III))	Activated during *in vitro* and *in vivo* transdifferentiation	Kristensen et al., 2000
*COLEC11* (Collectin-11)	Identified by proteomic functional comparative hotspot analysis of liver cells for genes associated with ECM assembly	Azimifar et al., 2014
*COMT* (Catechol-o-methyltransferase)	Activated during *in vitro* and *in vivo* transdifferentiation	Kristensen et al., 2000
*CRTAP* (Cartilage-associated protein)	Activated during *in vitro* and *in vivo* transdifferentiation	Kristensen et al., 2000
*CSPG4* (Chondroitin, Chondroitin sulphate proteoglycan)	Activated HSCs grown on plastic produce more chondrotin than HSCs grown on a basement membrane-like matrix	Friedman et al., 1989
*CSRP2* (cysteine and glycine-rich protein 2)	In liver, the gene *CSRP2* is exclusively expressed by stellate cells, whereas no transcripts are detectable in hepatocytes, sinusoidal endothelial cells or Kupffer cells	Weiskirchen et al., 2001
*CTSD* (Cathepsin D)	Activated during *in vitro* and *in vivo* transdifferentiation	Kristensen et al., 2000
*CYGB* (Cytoglobin, STAP, stellate cell activation-associated protein)	STAP is dramatically induced in *in vivo* activated HSCs isolated from fibrotic liver and in HSCs undergoing *in vitro* activation during primary culture	Kawada et al., 2001; Kawada, 2015
*DCN* (decorin)	The steady-state levels of the mRNA for decorin increased fourfold during primary HSC culture	Meyer et al., 1992; Ji et al., 2012
*DPT* (Dermatopontin)	Identified by proteomic functional comparative hotspot analysis of liver cells for genes associated with ECM assembly	Azimifar et al., 2014
*DES* (desmin)	Desmin increases during culturing in rat HSCs	Ramadori et al., 1990
*EDSP1* (Dermatan sulphate proteoglycan)	Activated HSCs grown on plastic produce more dermatan than HSCs grown on a basement membrane-like matrix	Friedman et al., 1989
*ENO2* (γ enolase)	Activated during *in vitro* and *in vivo* transdifferentiation	Kristensen et al., 2000
*FBLN5* (Fibulin-5)	Identified by proteomic functional comparative hotspot analysis of liver cells for genes associated with ECM assembly	Azimifar et al., 2014
*FDPS* (Farnesyl pyrophosphate synthetase)	Activated during *in vitro* and *in vivo* transdifferentiation	Kristensen et al., 2000
*GAL1* (galectin-1)	Activated during *in vitro* and *in vivo* transdifferentiation	Kristensen et al., 2000; Ji et al., 2012
*GFAP* (glial fibrillary acidic protein)	GFAP is a cell type specific marker for HSCs	Neubauer et al., 1996
*GKN2* (Gastokine-2)	Identified by proteomic functional comparative hotspot analysis of liver cells for genes associated with ECM assembly	Azimifar et al., 2014
Heparan (*HSPG2*)	Activated HSCs grown on plastic produce more heparan than HSCs grown on a basement membrane-like matrix	Friedman et al., 1989
*KLF6* (Kruppel-like factor 6, ZF9, zinc finger transcription factor 9)	Both the expression and biosynthesis are increased markedly in activated HSCs *in vivo* compared with quiescent HSCs	Ratziu et al., 1998
*LOXL2* (Lysyl oxidase-like 2)	Identified by proteomic functional comparative hotspot analysis of liver cells for genes associated with ECM assembly	Azimifar et al., 2014
*MMP2* (72 kDa type IV collagenase, MMP 2)	Activated during *in vitro* and *in vivo* transdifferentiation	Kristensen et al., 2000
*MYH11* (smooth muscle myosin heavy chain 11)	Activated rat HSCs express this smooth muscle cell marker	Wirz et al., 2008
*MYOCD* (Myocardin)	Activated rat HSCs express this smooth muscle cell marker	Wirz et al., 2008
*NCAM1* (Neural cell adhesion molecule 1, CD56)	Rat HSCs specifically express N-CAM and expression is activated during *in vitro* and *in vivo* transdifferentiation	Knittel et al., 1996a; Kristensen et al., 2000
*NES* (nestin)	The neural stem cell marker nestin is induced during activation of rat hepatic stellate cells	Niki et al., 1999
*NGF* (nerve growth factor)	HSCs show immunoreactivity for the neutrophin NGF	Cassiman et al., 2001
*NGFR* (p75(NTR), TNFRSF16, CD271, nerve growth factor receptor)	Activated HSCs express p75(NTR)	Trim et al., 2000; Cassiman et al., 2001
*NTF3* (NT3, neurotrophin 3)	HSCs show immunoreactivity for the neurotrophin 3	Cassiman et al., 2001
*NTF4/5* (NT4/5, neurotrophin 4/5)	HSCs show immunoreactivity for the neurotrophin 4/5	Cassiman et al., 2001
*NTRK2* (neurotrophic tyrosine kinase receptor type 2, Trk-B, Tyrosine kinase receptor B)	HSCs show immunoreactivity for the tyrosine kinase receptors (Trk) B	Cassiman et al., 2001
*NTRK3* (neurotrophic tyrosine kinase receptor type 3, Trk-C, Tyrosine kinase receptor C)	HSCs show immunoreactivity for the tyrosine kinase receptors (Trk) C	Cassiman et al., 2001
*P4HA2* (Prolyl 4-hydroxylase α)	Activated during *in vitro* and *in vivo* transdifferentiation	Kristensen et al., 2000
*PAI1* (Plasminogen activator inhibitor 1)	PAI-1 production in HSCs is stimulated by TGF-β	Knittel et al., 1996b
*PCDH7* (Protocadherin 7)	Identified as a HSC-specific surface marker	Zhang et al., 2015^∗∗∗^
*PDGFRA* (PDGFRα, platelet-derived growth factor receptor α)	PDGFRα is primarily expressed in HSCs, and *Pdgfrα* expression increased in injured mouse livers	Hayes et al., 2014
*PDGFRB* (PDGFRβ, platelet-derived growth factor receptor β)	PDGFRβ mRNA and protein were induced in response to TGF-β1 in human HSCs	Pinzani et al., 1995
*PLA2R1* (PLA2R, phospholipase A2 receptor)	Identified by proteomic functional comparative hotspot analysis of liver cells for genes associated with ECM assembly	Azimifar et al., 2014
*PRNP* (Prion-related protein PrP^C^)	PrP expression is closely related to stellate cell activation	Ikeda et al., 1998
*RBP1* (CRBP1, cellular retinol-binding protein)	CRBP-1 expression gradually increase during culture activation of HSCs	Uchio et al., 2002; Ji et al., 2012
*S100A6* (Calcyclin, S100 calcium binding protein A6)	Activated during *in vitro* and *in vivo* transdifferentiation	Kristensen et al., 2000
*S100A11* (Calgizzarin, Calcium-binding protein A11)	Activated during *in vitro* and *in vivo* transdifferentiation	Kristensen et al., 2000
*SLC2A13* (Hmit, solute carrier family 2)	Identified by proteomic functional comparative hotspot analysis of liver cells for genes associated with ECM assembly	Azimifar et al., 2014
*SMOC2* (SPARC-related modular calcium-binding protein 2)	Identified by proteomic functional comparative hotspot analysis of liver cells for genes associated with ECM assembly	Azimifar et al., 2014
*SPARC* (Secreted protein, acidic, cysteinee-rich, osteonectin, ON, BM40)	Activated during *in vitro* and *in vivo* transdifferentiation	Kristensen et al., 2000
*SPP1* (Secreted phosphoprotein 1, Osteopontin)	Osteopontin is significantly increased during the progressive activation of cultured rat HSCs and induced during experimental hepatic fibrosis	Lee et al., 2004
*SYP* (Synaptophysin)	Synaptophysin is a marker for quiescent as well as activated human and rat HSCs	Cassiman et al., 1999
*TAGLN* (Transgelin, SM22α)	The SM22α promoter is sufficient to achieve strong or partially selective expression *in vitro* but is not able to direct a specific or inducible expression of transgenes in HSCs/MFBs *in vivo*	Herrmann et al., 2004
*TGFA* (TGF-α, transforming growth factor α)	During transdifferentiation MFBs increasingly express TGF-α	Gressner, 1996
*TGFB1* (TGF-β1, transforming growth factor-β1)	During transdifferentiation MFBs increasingly express TGF-β1	Gressner, 1996
*TGFB3* (TGF-β3, transforming growth factor-β3)	Induced in rat HSCs during culture activation	Ji et al., 2012
*TIMP1* (Tissue inhibitor of metalloproteinase 1)	TIMP-1 expression is upregulated in culture-activated rat HSCs and rat models of liver injury and fibrosis	Iredale et al., 1996
*TMOD2* (Tropomodulin 2)	Identified by proteomic functional comparative hotspot analysis of liver cells for genes associated with ECM assembly	Azimifar et al., 2014
*TNA* (Tetranectin)	Identified by proteomic functional comparative hotspot analysis of liver cells for genes associated with ECM assembly	Azimifar et al., 2014
*VIM* (vimentin)	Vimentin increases during culturing in rat HSCs	Ramadori et al., 1990


*^∗^In this study, 21 proteins/polypeptides were identified that are transdifferentiation-dependent upregulated *in vitro* and *in vivo*, and a couple of proteins that become differentially activated during both experimental settings. ^∗∗^This proteome study identified 319 rat proteins that were significantly upregulated during HSC activation from which 126 were already identified in a previous study (Kristensen et al., 2000). ^∗∗∗^This study compared HSCs, immune and hepatic transcriptome profiles and defined a 122-gene HSC signature.*

The identification of factors and *cis*- or *trans*-acting elements within such genes that trigger preferred expression in HSCs/MFBs offer important therapeutic opportunities. Such regulatory elements could be exploited to express antifibrotic or apoptosis-associated transgenes specifically in physiologically altered HSCs, thereby inducing targeted clearance of reprogrammed, activated HSCs/MFBs or preventing hepatic expression of excessive ECM constituents. Several years ago, we have shown in culture-activated HSCs that promoter fragments of the *CSRP2* and *SM22α* genes that are expressed exclusively in the liver in HSCs are sufficient to mediate reporter gene expression (Herrmann et al., 2004). Similarly, we found that stretches taken from the transdifferentiation-sensitive *TIMP-1* promoter were able to achieve transgene expression in culture-activated HSCs but not in cultured hepatocytes (Herrmann et al., 2004). In a similar targeted gene transcription approach it was shown that a 2.2-kbp fragment derived from the human *GFAP* gene promoter was not only capable of directing HSC-specific expression *in vitro*, but also conferred dose- and time-dependent sensitivity to TGF-β that is the major cytokine responsible for ECM formation (Maubach et al., 2006).

In another investigation, the *GFAP* promoter was fused to the herpes simplex virus thymidine kinase gene. This transgene was able to render susceptibility for ganciclovir-induced cell death, both in *in vitro* and *in vivo* (Puche et al., 2013). However, the usage of the *GFAP* promoter as a regulatory element to drive transgene expression in activated HSCs or transdifferentiated MFBs is potentially hindered by the finding that HSCs downregulate expression of *GFAP* and that this gene is also significantly expressed in neuronal tissues and cholangiocytes (Yang et al., 2008). A similar finding was noticed for the vimentin promoter that is predominantly active in activated HSCs and MFBs in the liver, but is also effectively transcribed in vascular smooth muscle cells and portal fibroblasts (Troeger et al., 2012).

Unfortunately, the partial cellular selectivity of other promoters that we observed *in vitro* was not found *in vivo* emphasizing the complexity of regulatory factors that are necessary to guarantee specific expression in HSCs (Herrmann et al., 2004). In addition, the detailed analysis of α-SMA and collagen I gene transcriptional patterns in primary cultures of HSCs and in an *in vivo* model of secondary biliary fibrosis by means of transgenic mice that express the red fluorescent protein and the enhanced GFP under transcriptional control of respective promoter elements revealed that these two genes were not co-expressed in all cells (Magness et al., 2004). These data indicate that HSCs are a fraction of cells that show heterogeneity during hepatic insult, thereby potentially hampering and restricting target approaches that use “HSC-specific” or promoters that become activated during hepatic insult.

## miRNA-Based Strategies for HSC Targeting

**FIGURE 8:**
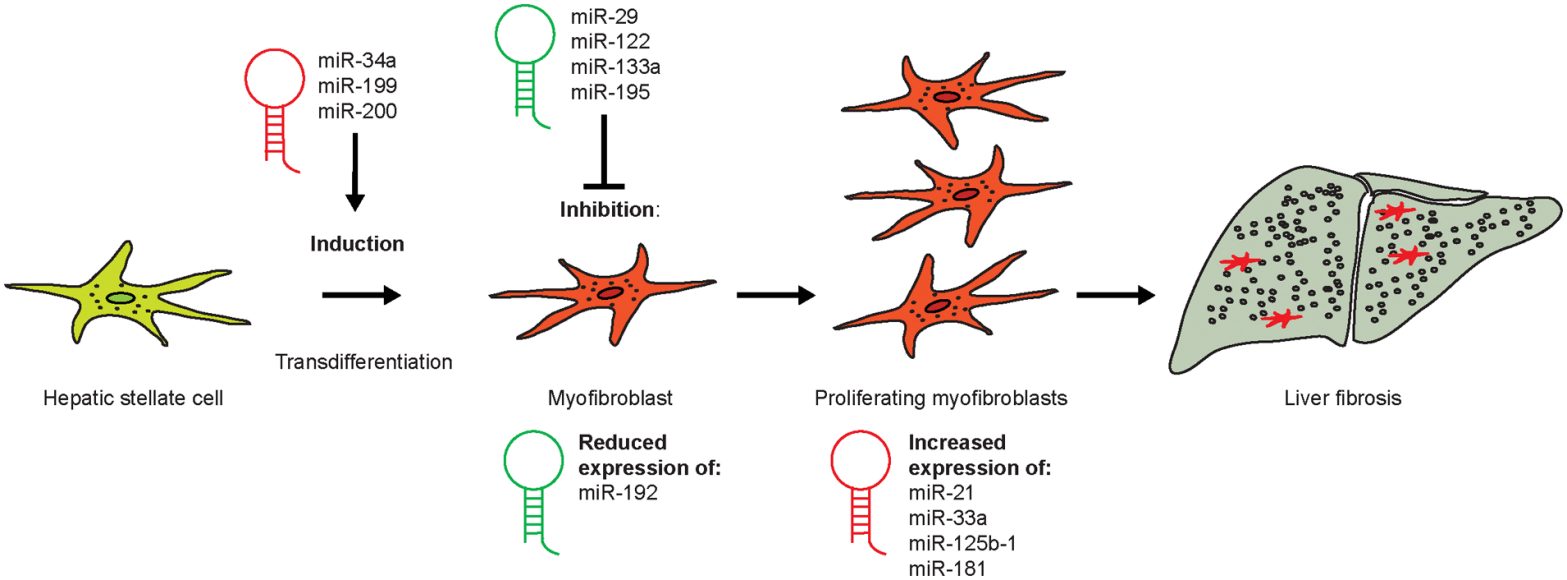
**Role of different micro-RNA during the process of HSC transdifferentiation in liver fibrosis.** Quiescent HSCs can become activated by certain miR during early fibrogenesis (miR-34a, -199, and -200), whereas the administration of others (e.g., miR-29, -122, and -195) might represent novel treatment options for liver fibrosis. miR-192 expression is strongly reduced in early stages of HSC activation, whereas those miR that increase in HSCs during fibrosis (miR-21, -33a, -125b-1, and -181) represent putative therapeutic targets in fibrosis therapy.

Micro-RNAs (miR) are small but powerful molecules, which usually size around 20 nucleotides and affect the regulation of mRNA (Lam et al., 2015). miR act by binding to the 3′ untranslated region (3′ UTR) of the target mRNA (Lam et al., 2015). Since the complementarity of miR-to-mRNA sequence is only partial, the miR can bind to multiple different mRNAs (Lam et al., 2015). Bioinformatics-based predictions have led to the assumption that about 60% of protein coding genes are regulated by miR (Friedman et al., 2009). Many different miR are involved in inflammatory diseases (Roy et al., 2015). Inflammation is considered the main initiating event in fibrogenesis and an important driver of fibrosis. During inflammation and fibrogenesis, HSCs undergo transdifferentiation into activated MFBs accompanied with cell proliferation. Several different miRs have specific roles during the process of HSC transdifferentiation in liver fibrosis (**Figure 8**).

Moreover, miR-34a and -199 play pivotal roles in the initiation of HSC transdifferentiation (Roy et al., 2015). miR-34a, which targets ASCL1, is upregulated during the initiation of liver fibrosis in a model of DMN-induced fibrosis (Li et al., 2011). The upstream target of miR-34a is the tumor suppressor p53 that can initiate cell cycle arrest, which explains the effects on HSC proliferation (Okada et al., 2014). Serum levels of miR-34a were also reported to be increased in patients with NAFLD (Cermelli et al., 2011). A hallmark study identified key miRs using miR arrays by comprehensively studying experimental fibrosis models in mice (Roderburg et al., 2011). Among these miRs, miR-29b is part of a signaling nexus involving TGF-β- and NF-κB-dependent signals in HSCs (Roderburg et al., 2011), while miR-133a mediates TGF-β-dependent derepression of collagen synthesis in HSCs (Roderburg et al., 2013). These and other studies suggested circulating miR may be potentially a novel diagnostic biomarker for liver fibrosis progression (Roderburg et al., 2012). For instance, members of the miR-199 and miR-200 family, namely miR-199a, -199a^∗^, -200a, and -200b, correlated with the progression of liver fibrosis both in murine models and human patients (Murakami et al., 2011).

Besides being critical initiators of HSC activation and fibrogenesis, there are also miR, which exhibit therapeutic potential due to their inhibiting effects on HSC disease progressing activation and which can therefore be designated as antifibrotic. The miR-29 was reported to down-regulate ECM synthesis in mouse models and human patients by among others targeting several collagen-associated genes (Bandyopadhyay et al., 2011). Characterized by the sole inhibition of *P4HA1*, the liver-specific miR-122 was found to reduce proliferation and activation of HSCs (Li et al., 2013; Shah et al., 2013). Down-regulation of cyclin E1 was shown for miR-195, and thereby, proliferation of HSCs was reduced in humans (Sekiya et al., 2011). Recently, miR-192 was identified as an important molecule in the transdifferentiation process of HSCs by Coll et al. (2015). It was strongly downregulated in cirrhotic livers compared to healthy organs as an early event during fibrosis progression and had a large number of 28 predicted target genes, based on an analysis of human HSCs. The authors found miR-192 to be strongly downregulated (nearly abrogated) already during early CCl_4_-based liver fibrosis (Coll et al., 2015). These observations put miR-192 into the role of a promising biomarker or target for early fibrosis.

After having progressed into activated, proliferating HSCs, the cells were observed to express a variety of miR indicative for their activation status. These molecules represent potential molecular targets for interventions, such as miR21, which modulates ERK1 signaling in HSC activation and the EMT of hepatocytes *via* inhibiting *SPRY2* and *HNF4α* mRNA (Zhao et al., 2014). miR-33a particularly increases during the progression of human and murine liver fibrosis in HSCs only. Toward their stimulation with TGF-β, HSCs react with upregulation of miR-33a, a regulator of lipid and cholesterol metabolism, which probably acts through Smad7 (Huang et al., 2015). The expression of miR125b-1 was described as being significantly increased in HSCs of liver fibrosis patients (Coll et al., 2015). miR-181b, which activates HSCs based on the phosphatase and tensin homolog deleted on chromosome 10 (PTEN)/Akt pathway, and further correlates with human liver disease progression (Yu et al., 2015).

## Clinical Translation of Preclinical Findings

Despite numerous innovative anti-fibrotics in preclinical settings, the number of those entering clinical studies is limited. At present, the best option for fibrosis treatment remains in eliminating the underlying cause of liver disease. For instance, antiviral therapies in virus hepatitis, alcohol abstinence or lifestyle changes represent effective antifibrotic measures in the respective liver diseases (Tacke and Trautwein, 2015).

Many preclinical studies suffer from the drawback that they focus on single cells or molecules, which help to identify mechanisms but ignore the complexity of biological systems. For instance, IFN-α or IFN-γ act as efficient anti-fibrotics *in vitro* (Mallat et al., 1995), but were not successful in the clinics (Fernández et al., 2006; Di Bisceglie et al., 2008). Earlier attempts to neutralize TGF-β suffer from a large number of effects of the cytokine on other cell types when administered parenterally. Therefore, specificity is an important issue, which has to be addressed more properly. For this aim, nanotechnological and innovative formulations, which specifically target HSC-expressed receptors such as the Rho kinase inhibitor Y27632 coupled to mannose-6-phosphate, may point toward novel directions (van Beuge et al., 2011).

Despite the fact that the existence of fibrosis regression represents a major source of hope for the success of novel treatment options, it has to be considered further that many of the standard models of hepatic rodent fibrosis, such as CCl_4_-mediated liver fibrosis, is reversible, which is in big contrast to human liver cirrhosis. It is therefore advisable that rodent models with little degree of regression should be used as well. Additionally, the time frame for human fibrogenesis usually comprises years but not weeks. Nevertheless, new compounds targeting such “late events” are currently under investigation. The antibody simtuzumab antagonizes LOXL2, which is an enzyme that cross-links collagen fibers as a late event in fibrogenesis (Barry-Hamilton et al., 2010).

Another challenge is the design of proper clinical trials, including suitable endpoints for such trials. At the moment, most trials rely on serial liver biopsies. Novel biomarkers, which allow specific fibrosis staging, and other non-invasive methods are promising novel directions for monitoring the success of antifibrotic therapies (Schuppan and Pinzani, 2012). Progresses in HSC targeting with nanotechnological formulations or innovative molecules such as miR might help to finally bring novel personalized therapeutic strategies to the clinics (Schuppan and Pinzani, 2012).

## Ethics Statement

The analysis of human samples (**Figure 2**) was approved by the Institutional Review Board of the Bonn University Ethics Committee (decision #067/10). Isolation of primary rat liver cells (**Figure 5**) and application of antifibrotic gene devices (**Figure 4**) was approved by the Bezirksregierung Köln (Cologne, Germany) and the Landesamt für Natur, Umwelt und Verbraucherschutz Nordrhein-Westfalen (Recklinghausen, Germany).

## Author Contributions

H-TS, MB, TL, FT, and RW have written this review. EB-K performed the experiments depicted in **Figure 2**. JN provided the human samples and requested the necessary ethic vote for the experiments shown in **Figure 2**. All authors have read this review and gave their agreement for submission.

## Conflict of Interest Statement

The authors declare that the research was conducted in the absence of any commercial or financial relationships that could be construed as a potential conflict of interest.

## Abbreviations

α-SMA: α-smooth muscle actin
Ad: adenovirus
BDNF: brain-derived growth factor
BiPPB: bicyclic PDGFβR-binding peptide
BSA: bovine serum albumin
CAR: coxsackievirus-adenovirus receptor
CCl_4_: carbon tetrachloride
CREBBP: CREB binding protein
CTGF: connective tissue growth factor
DMN: dimethylnitrosamine
DOX: doxorubicin
ECM: extracellular matrix
EMT: epithelial-to-mesenchymal transition
GFP: green fluorescent protein
HCC: hepatocellular carcinoma
HGF: hepatocyte growth factor
HSA: human serum albumin
HSC(s): hepatic stellate cell(s)
IFN-γ: interferon-γ
IL10: interleukin-10
KC(s): Kupffer cell(s)
LAP: latency-associated peptide
LOXL2: lysyl oxidase like 2
LSEC(s): liver sinusoidal endothelial cell(s)
M6P: mannose 6-phosphate
M6P/IGFII: mannose 6-phosphate/insulin-like growth factor II
MFB(s), myofibroblast(s), mim γ, IFN-γ, peptidomimetic, miR: miRNA(s)
MMP: matrix metalloproteinase
MPA: mycophenolic acid
NGF: nerve growth factor
MSCs: mesenchymal stem cells
NAFLD: non-alcoholic fatty liver disease
NASH: non-alcoholic steatohepatitis
NT: neurotrophin
OM: Oxymatrine
p75NTR: p75 neurotrophin receptor
PDGF: platelet-derived growth factor
PDGFβR: PDGF receptor type β
PEG: polyethylene glycol
PLGA: poly(lactic-co-glycolic acid)
PPARγ: peroxisome proliferator-activated receptor γ
PPB: PDGFβR-recognizing cyclic peptide
PTEN: phosphatase and tensin homolog
PTX: pentoxifylline
RBP: retinol-binding protein
SSLs: sterically stable liposomes
TFO(s): triplex forming oligonucleotide(s)
TGF: transforming growth factor
TIMP: tissue inhibitor of metalloproteinase
TNF: tumor necrosis factor
Trk: tropomyosin-related kinase
TSP-1: thrombospondin-1
